# Microfluidic Platform for the Long-Term On-Chip Cultivation of Mammalian Cells for Lab-On-A-Chip Applications

**DOI:** 10.3390/s17071603

**Published:** 2017-07-10

**Authors:** Frank Bunge, Sander van den Driesche, Michael J. Vellekoop

**Affiliations:** 1 Institute for Microsensors, -actuators and -systems (IMSAS), University of Bremen, 28359 Bremen, Germany; sdriesche@uni-bremen.de (S.v.d.D.); mvellekoop@imsas.uni-bremen.de (M.J.V.); 2 Microsystems Center Bremen (MCB), University of Bremen, 28359 Bremen, Germany

**Keywords:** Lab-on-a-Chip, hydrogel, cell cultivation, MDCK, diffusion model, parylene

## Abstract

Lab-on-a-Chip (LoC) applications for the long-term analysis of mammalian cells are still very rare due to the lack of convenient cell cultivation devices. The difficulties are the integration of suitable supply structures, the need of expensive equipment like an incubator and sophisticated pumps as well as the choice of material. The presented device is made out of hard, but non-cytotoxic materials (silicon and glass) and contains two vertical arranged membranes out of hydrogel. The porous membranes are used to separate the culture chamber from two supply channels for gases and nutrients. The cells are fed continuously by diffusion through the membranes without the need of an incubator and low requirements on the supply of medium to the assembly. The diffusion of oxygen is modelled in order to find the optimal dimensions of the chamber. The chip is connected via 3D-printed holders to the macroscopic world. The holders are coated with Parlyene C to ensure that only biocompatible materials are in contact with the culture medium. The experiments with MDCK-cells show the successful seeding inside the chip, culturing and passaging. Consequently, the presented platform is a step towards Lab-on-a-Chip applications that require long-term cultivation of mammalian cells.

## 1. Introduction

Lab-on-a-chip (LoC) is a vision that was developed decades ago with the start of the first microfluidic devices. LoC means that an entire laboratory analysis process is integrated on one microfluidic chip that ideally runs automated, cheaper and faster than the traditional process. Consequently, such an LoC has to manage the handling of the fluidic samples, the preparation, the analysis and the interpretation of the data. In recent years, it has become clear that such a chip would be extremely complex and thus not realistic. Therefore, the idea of “chip in a lab” was developed, where the microfluidic chip contains only a few functionalities but require external equipment. However, the extensive use of such equipment hampers the use of “chip in a lab” as conventional methods might be still cheaper and/or easier to use. Therefore, “chip in a lab” will only be successful if a better trade-off between the complex integration of functionalities and expensive external equipment is found, which will require new concepts of microfluidic chips.

Nevertheless, some LoCs have already found their way into the market like blood analysis or detection of infections [1]. These systems have in common that the analysis is carried out over short time periods and are not based on the cultivation of mammalian cells. However, such cell culturing devices are also attractive for LoC because sensing elements could be included [2,3,4], the cells could be cultured in a 3D-matrix [5,6,7,8], or different cell types could be combined to form organs-on-a-chip [9] or even a body-on-a-chip [10,11].

Basically, mammalian cells require regularly fresh medium, a constant supply of oxygen (and carbon dioxide for the sodium bicarbonate buffer) and a suitable environment (constant temperature, no exposure to cytotoxic materials, usually no shear stress). Ideally, an LoC for long-term cell cultivation integrates the two supplies and provide such an environment without external equipment.

The supply with nutrition is realized either by a relatively large reservoir in an open chip [12] or by channels inside the chip. Open assemblies also solve the gas supply if they are placed in an incubator but are prone to infections and provide only a low level of system integration resulting in high assembly costs and high operational costs. The supply in a closed microfluidic chip is either perfusion based, where the liquid flows above the cells, or diffusion based through a membrane. Perfusion results in a constant supply even at large structures but also in shear stress on the cells [13]. Such stress influences the growth of the cells, which might be a desired effect but might also disturb the culture [14,15]. Obviously, a very low flow rate minimizes this influence but requires a sophisticated pump.

Diffusion based culture devices avoid this stress completely. Here, the supply of nutrition is realized by a porous membrane under the cell culture ( horizontal membrane) [2] or next to the cell culture (vertical membrane) [16]. Horizontal membranes are attractive because the diffusion rates are usually high and equally distributed. However, the integration of such a membrane e.g., made out of polycarbonate [2,17], is not compatible with standard processes and thus hard to fabricate at reasonable costs. The vertical membranes are usually made out of hydrogel that is melted when it is inserted into the chip and gels to form the membrane. Here, the difficulty is to fill only the desired parts of the chip and keep the nutrition channel and the culture chamber free of gel. This problem is usually solved by an array of micropillars [7,16] that is filled prior to the rest of the chip. However, these pillars reduce the effective cross-section area for the diffusion and thus limit the supply. Furthermore, the width of culture chamber is also limited by the diffusion rates, which hinders the up-scaling of such a system.

The supply with gases is realized either by dissolving the gases in the culture medium [18] or by diffusion through the chip material [19]. The first strategy means on one hand that the choice of the material is not limited to gas permeable materials. On the other hand, the gas and nutrition supply are coupled and constant pumping is required. Furthermore, the setup to dissolve the gases is bulky, usually external and thus do not fulfill the idea of LoC. Therefore, the vast majority of the microfluidic cell culture devices use permeable materials and here especially polydimethylsiloxane (PDMS). PDMS has some major advantages regarding the rapid prototyping. The molding process is relatively fast, low cost and PDMS can be bonded easily to glass or another layer of PDMS. Furthermore, PDMS is attractive because the structures for the gas supply are omitted. Here, the oxygen and carbon dioxide might diffuse directly from the environment to the cells but that requires an external incubator which is not in the sense of LoC. Another method is the integration of gas channels [20,21,22], which are connected to gas reservoirs of oxygen and carbon dioxide [23].

However, PDMS has also some serious disadvantages [24]. First of all, it is permeable to water vapor meaning that the osmolarity changes over time unless there is a constant flow of fresh liquid. Furthermore, PDMS absorbs small, usually hydrophobic molecules such as signal molecules or hormones (e.g., estrogen) [25]. In contrast to the absorption, small molecules might also leak into the liquid. This leakage tampers experimental results e.g., when proteins leak from PDMS of previous measurements but is also problematic for oligomers, which were not crosslinked to other molecules during the fabrication process of PDMS. These uncrosslinked oligomers are cytotoxic [25]. Additionally, PDMS changes its surface properties, e.g., recovering its hydrophobicity after an oxygen plasma treatment within a few days. PDMS is optical transparent but also autofluorescent and last but not least flexible. The flexibility is advantageous e.g., for the integration of valves or pumps [13] but problematic for e.g., mechanobiological experiments.

There are many microfluidic applications where these disadvantages are irrelevant. These applications are usually either short-term or based on constant exchange of the entire medium to level out the absorption and leakage. However, it is not suitable for long-term experiments with cell cultures [26]. Apart from that, PDMS also provides problems with scaling up and with fabrication for industrial use at large scale [27].

To sum it up, various publications in the past as listed in Table 1 proved the attractiveness of LoC for cell cultivation purposes. However, the current microfluidic chips still provide some disadvantages, mainly the use of PDMS as chip material and difficulties with the supply of nutrition, which hamper the breakthrough.

Previously, we introduced a concept for the on-chip cell cultivation and showed the first experimental results [28]. This concept is based on integration of vertical, porous membranes out of hydrogel through which gases and nutrition diffuse from individual channels. Apart from the hydrogel membranes, the chip is made out of non-cytotoxic, hard materials by standard clean room processes. Here, we discuss this concept in detail, especially the dimensioning of the chip for sufficient oxygen supply, the assembly and the experimental results.

## 2. Design Aspects

### 2.1. Concept

**Figure 1:**
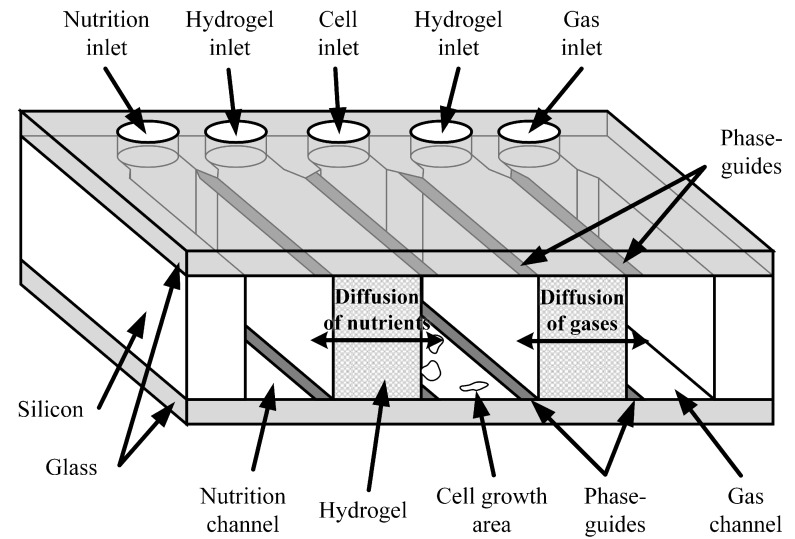
Concept of the microfluidic chip for the long-term cultivation of mammalian cells in a lab-on-a-chip context as a cross-section. The half of the chip that is cut away for better visualisation is identical to the shown one. The figure is not to scale.

The chip consist of a large cultivation chamber and two microfluidic channels (see Figure 1).

One channel is used to supply fresh medium and the other one for the gas supply. The channels and the chamber are separated from each other by vertical walls out of hydrogel which is porous and thus permeable for gases and nutrition. Consequently, the supply to the cells is based solely on diffusion, meaning that any shear stress on the cells is avoided. The integration of the hydrogels is based on the application of surficial phaseguides as shown in previous work [29]. Here, the melted hydrogel is pulled into the chip by the negative capillary pressure between the hydrophilic surfaces of the glass. The undesired filling of the culture chamber and the supply channels is avoided by patterning hydrophobic, flat elements (referred to as phaseguides) on the top and bottom plate. As these several nm high elements are hydrophobic, the melted hydrogel does not flow over them unless high pressure is applied. Thus, the hydrogel membranes are integrated without bulky elements like geometrical phaseguides [30] or pillars [7,16], resulting in a maximal cross-section area and thus maximal diffusion rates.

### 2.2. Design of the Hydrogel Membranes

The hydrogel membranes are fabricated by gelation of the melted gel in predefined regions inside the chip. These regions are surrounded by the hydrophobic phaseguides that are flat elements on the bottom and top of the chip. In order to avoid the gel flowing over the phaseguides, the filling should be achieved pressureless and thus by benefiting of the negative capillary pressure between the hydrophilic glass plates. The aspect ratio of the wall-less channel depends on the contact angle of the glass but is always below 1 [29]. Technically speaking, a reliable filling is achieved if the channel is two to three times wider than high (i.e., aspect ratio 0.5 or 0.33). Wider channels are even more robust but lower the diffusion rates due to a high diffusion distance.

### 2.3. Dimensioning of the Culture Chamber

On one hand, the cultivation chamber should be designed as large as possible to enable the growth of a high number of cells. On the other hand, the supply is based on diffusion, meaning that the supply rates are limited by diffusion distances and thus the chamber width.

The cell metabolism of mammalian cells consists of several steps. At first, glucose is converted anaerobically via pyruvate, which is sometimes also part of the culture medium, to lactate (so called glycolysis). Lactate is subsequently degradated to carbon dioxide. In cell cultures, the glycolysis step is the dominant metabolic pathway in contrast to the in-vivo behaviour, where the entire degradation of glucose to carbon dioxide takes place [31]. In the case of MDCK-cells, the glucose consumption is around 250 $\frac{\mathrm{amol}}{\mathrm{cell}\;s}$ [32] so that a common concentration of 4500 $\frac{\mathrm{mg}}{L}$ is consumed within 14 h assuming a cell density of $1\times 10^{6}\mathrm{cell}/mL$. At the same time, lactate is produced with a rate of 490 $\frac{\mathrm{amol}}{\mathrm{cell}\;s}$ and ammonium with a rate of 11 $\frac{\mathrm{amol}}{\mathrm{cell}\;s}$ [32]. Once the concentration of lactate reaches 20 mM, the cell growth is inhibited and the cells die for concentrations higher than 40 mM but also for ammonium concentrations above 2 mM [33]. Consequently, the metabolic products have to be removed within 11 and 50 h.

The degradation of lactate to carbon dioxide is an aerobic reaction and thus results in an oxygen uptake. For MDCK-cells, the oxygen uptake is 20 $\frac{\mathrm{amol}}{\mathrm{cell}\;s}$ [34]. The concentration of dissolved oxygen depends on the partial pressure on the ambient oxygen, the temperature and the ionic strength. For cell cultivation at 37 ${}^{\circ }C$, the maximum concentration is around 0.2 mM. Consequently, the entire oxygen is consumed within 2.7 h.

In comparison of the compounds (see Table 2), the oxygen has by far the lowest exchange time. As the diffusion coefficients are in the same range, the supply of oxygen by diffusion is the most critical parameter for the design and thus limit the width of the chamber.

The oxygen diffuses through the chamber based on the Fick’s second law that is extended by the uptake of oxygen:

(1) $$\frac{dc}{dt}=D\Delta c-v,$$

where *c* is the concentration, *D* the diffusion coefficient, Δ the Laplace operator and *v* the oxygen uptake. In the *y*-direction, which is parallel to the phaseguides, no gradient will occur. Furthermore, the chamber and the channels are much wider than high, the diffusion can be modelled one-dimensional (here: *x*-direction, i.e., perpendicular to the membranes) as

(2) $$\frac{dc}{dt}=D\frac{d^{2}c}{dx^{2}}-\frac{\gamma OCR}{A_{c}h},$$

where OCR is the oxygen consumption rate of each cell, γ describes the relative amount of surface that is covered with cells, $A_{c}$ is the area of each cell and *h* is the height of the chamber. In the stationary phase, the term $\frac{dc}{dt}$ can be neglected and the differential equation is solved to

(3) $$c\left(x\right)=\frac{\gamma OCR}{2A_{c}hD}x^{2}+k_{1}x+k_{2}.$$

x=0 is defined at the interface between the hydrogel towards the gas supply channel and the culture chamber. At the interface between the hydrogel and the gas channel, the oxygen concentration can be assumed as constant ($c\left(x=-w_{Hg}\right)=c_{0}$). In a worst-case-scenario, it can be also assumed that no oxygen diffuses from the nutrition channel towards the cells (e.g., if the culture medium does not contain oxygen or if the medium is not constantly pumped through) and so $\frac{dc(x=w_{Gr})}{dx}=0$. Here, $w_{Gr}$ describes the maximal width where cells survive which is either the maximal width of the culture chamber or the point without oxygen ($c(x=w_{Gr})=0)$.

The hydrogel membranes do not contain any cells and thus does not consume any oxygen. The oxygen concentration for the presented device with vertical membranes is found as

(4) $$c\left(x\right)=\left\{\begin{array}{cc} k_{1}x+k_{2} & -w_{Hg}\leq x<0\\ \frac{\gamma OCR}{2A_{c}hD}x^{2}+k_{1}x+k_{2} & 0\leq x<w_{Gr} \end{array}\right\},$$

with

(5) $$\begin{array}{ccc} k_{1} & = & \frac{-\gamma OCRw_{Gr}}{A_{z}hD}, \end{array}$$

(6) $$\begin{array}{ccc} k_{2} & = & c_{0}-\frac{\gamma OCRw_{Gr}w_{Hg}}{A_{c}hD}. \end{array}$$

**Figure 2:**
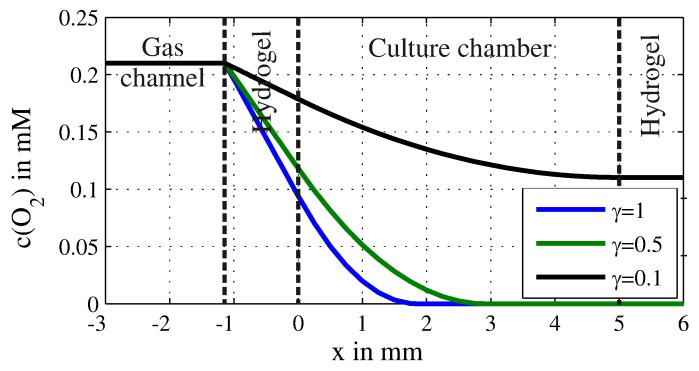
Course of the concentration of oxygen $c(O_{2})$ in the steady state is shown for $w_{Hg}=1.1$ mm, h=0.38 mm, $A_{c}=400\;µm^{2}$, $D=2.4\times 10^{-9}\;\frac{m^{2}}{s}$, a width of the culture chamber of 5 mm and different filling factors γ.

The oxygen concentration decreases linearly within the hydrogel membrane and parabolically within the cell culture. The course of the concentration is shown as exemplary in Figure 2 for $w_{Hg}=1.1$ mm, h=0.38 mm, $A_{c}=400\;µm^{2}$, $D=2.4\times 10^{-9}\;\frac{m^{2}}{s}$, a width of the culture chamber of 5 mm and different filling factors γ. For low filling factors, the entire chamber is supplied with oxygen, while, for γ=0.5, no more oxygen is available for x>3 mm.

These results are in good agreement with simulations based on finite-element methods (FEM) as presented earlier [28]. In the case of γ=0.5, the maximum difference between the simulated concentration and the calculated concentration is only 0.6% although the model is just one-dimensional and FEM-simulation two-dimensional, where the oxygen uptake is simulated on the bottom the chamber while the diffusion occurs at the entire height.

With Equation (4), the maximum width of the growth area where $c(w_{Gr})=0$ is found as

(7) $$w_{Gr}=-w_{Hg}+\sqrt{{w_{Hg}}^{2}+\frac{2c_{0}A_{c}hD}{\gamma OCR}}.$$

**Figure 3:**
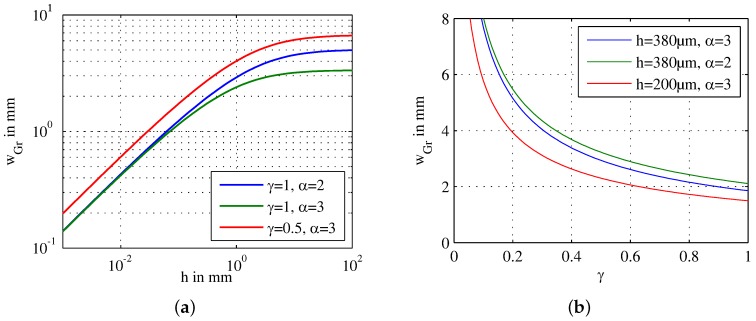
Maximal width of the growth chamber $w_{Gr}$ based on the analytical model: (**a**) width of the chamber as a function of the chamber height *h* for different filling factors γ and different α describing the ratio of the hydrogel width to the chamber height; (**b**) width of the chamber as a function of the filling factor γ, which describes the ratio of the surface that is covered with cells for different heights and α.

The width of the chamber depends on three design parameters:

1. $w_{Hg}$: Thinner hydrogel membranes result in wider chambers. Without any membrane ($w_{Hg}=0$), the maximum width is 3.9 mm. As mentioned above, the membrane is more robust if $\alpha =\frac{w_{Hg}}{h}=2...3$ (i.e., 0.8 mm and 1.1 mm), resulting in a width of the chamber of 3.2 mm and 2.9 mm, respectively.
2. *h*: The height has a major influence on the width of the growth chamber as shown in Figure 3a. Considering that the width of the hydrogel is proportional to the chamber height, there is an upper limit for the width of the growth chamber as
  (8) $$w_{Gr,\max}=\frac{c_{0}A_{c}D}{\alpha \gamma OCR}=6.7\mathrm{mm}.$$
  A good compromise between a maximal width of the growth chamber and easy fabrication is the usage of standard silicon wafers with a height of 380 µm for the walls. This results in a chamber width of 3.1 mm.
3. γ: The filling factor that describes how much area is covered with cells also has a major influence on the width of the growth area as shown in Figure 3b. Less cells obviously result in wider chambers. For γ=1, the width is reduced to 1.9 mm. However, mammalian cells need space to grow and proliferate in order to avoid stress on the cells. Consequently, limiting the filling factor to 0.5 is necessary for healthy cells, which means that the cells have to be split and a part of the cells have to be removed once 50% of the growth area is covered.

All mentioned values are calculated with $c_{0}=0.2\;mM$, $\alpha =\frac{w_{Hg}}{h}=3$, h=380 µm, γ=0.5, $A_{c}=400\;µm^{2}$, $OCR=20\times 10^{-18}\frac{\mathrm{mol}}{\mathrm{cell}\;s}$ and $D=2.4\times 10^{-9}\;\frac{m^{2}}{s}$ unless otherwise stated.

The final cultivation chamber is 0.38 mm high, 3 mm wide and 8 mm long, which results in a surface area of 24 ${\mathrm{mm}}^{2}$ and thus approximately 30,000 cells with a maximum filling factor of γ=0.5. Considering the volume of the chamber of 9.1 µL, the maximum cell density is $3.3\times 10^{6}\;\frac{\mathrm{cell}}{\mathrm{mL}}$.

## 3. Materials and Methods

### 3.1. Materials

The cell culture medium consists of Glasgow Minimum Essential Medium (GMEM) purchased from Sigma Aldrich (G5154) (St. Louis, Missouri, USA) to which 10% *w/w* fetal bovine serum and 1% *w/w* Penicillin-Streptomycin are added (Sigma Aldrich, St. Louis, Missouri, USA). The detaching of the MDCK-cells is done with TrypLE^TM^ Select Enzym (1x) purchased from ThermoFisher (Waltham, Massachusetts, USA). Agarose with a low gelling temperature (A9414) with a transition temperature between 26 and 30 ${}^{\circ }C$ was purchased also from Sigma Aldrich.

### 3.2. Fabrication

**Figure 4:**
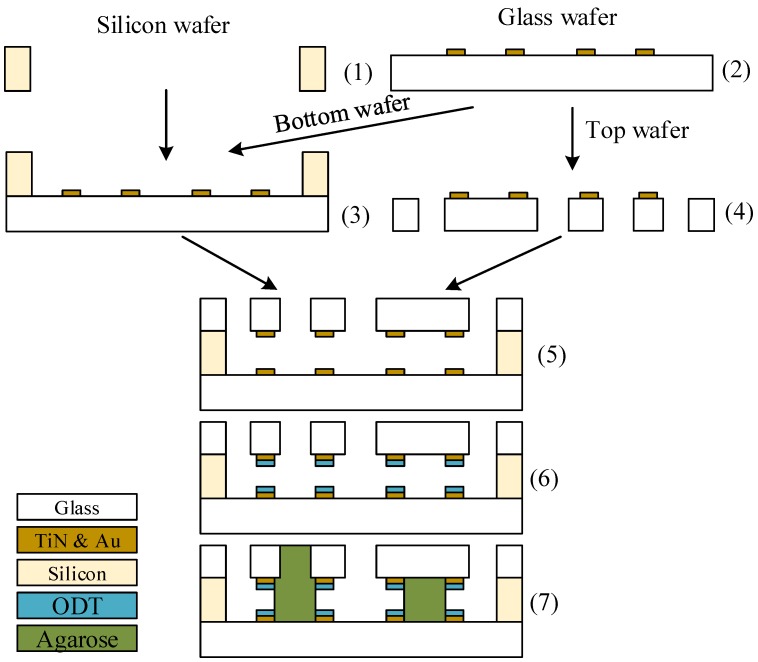
Fabrication of the device: (1) Deep reactive ion etching (DRIE) process for the channels into 380 µm thick silicon; (2) patterning of titanium nitride and gold on two 520 µm thick borosilicateglass wafers; (3) anodic bonding of one glass wafer and the silicon wafer; (4) powderblasting of the inlets into the second glass wafer; (5) anodic bonding; (6) coating of gold with octadecanethiol (ODT); (7) creation of the agarose membranes.

The presented device consists of three layers: the bottom layer out of borosilicate glass with the phaseguides out of coated gold, the middle layer which define the microfluidic channels and the top layer with the phaseguides out of gold and the inlets. The fabrication process is summarized in Figure 4.

The microfluidic channels are etched at first into a 380 µm thick, standard silicon wafer by a deep reactive ion etching (DRIE) process. Two glass wafers are coated with 10 nm titanium nitride and 40 nm gold. The titanium nitride increases the adhesion of the gold on the glass but does not impurify the gold by diffusion unlike other, more common adhesion promoters like chromium or titanium. The gold is patterned by photolithography and wet-etching with Au Etch 200 of NB Technologies GmbH, Bremen, Germany and the titanium nitride with a mixtures of 1 M HCl and 1.8 M H_2_O_2_.

**Figure 5:**
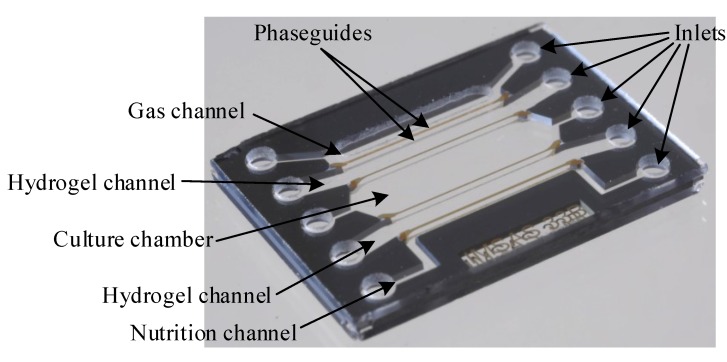
Image of the fabricated chip. The size of the chip is 13 × 17 × 1.4 mm${}^{3}$.

Subsequently, one glass wafer and the silicon wafer are bonded together by anodic bonding. The phaseguides out of gold slightly overlap with the silicon structures to compensate for any alignment and fabrication tolerances. Gold and silicon form an eutectic layer at temperatures above 363 ${}^{\circ }C$ that might damage the phaseguides. Consequently, the bonding is processed at 360 ${}^{\circ }C$ with a voltage of 600 V. The second glass wafer is powderblasted to form the inlets and outlets after laminating the mask out of i-HE dry film resist of Harke GmbH, Germany. Subsequently, this wafer is also bonded anodically onto the other side of the silicon wafer. Due to the isolation of the other wafer, this second bonding process is run at higher voltages of 1100 V. Finally, the wafer stack is diced and each chip (see Figure 5) is filled for 2 min with ethanol containing 1 $\frac{g}{L}$ octadecanethiol (ODT). ODT forms a self-assembled monolayer on gold but neither on silicon nor on glass. This monolayer is hydrophobic with a contact angle of $103\pm 1^{\circ }$ while the contact angle on glass is only $16\pm 2^{\circ }$.

### 3.3. Assembly

**Figure 6:**
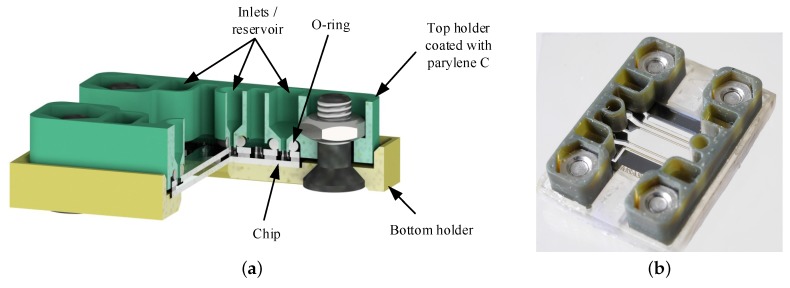
Assembly of the microfluidic chip that is clamped between 3D-printed holders and sealed with O-rings: (**a**) model of the assembly showing all components (**b**) image of the assembly with the dimensions of 29 × 21.7 × 7.9 mm.

The interface between the microfluidic chip and the macroscopic world is realized with a 3D-printed holder structure as shown in Figure 6. The holder consists of a bottom plate printed with the resin “Clear01” in the “Form 1” printer of Formlabs Inc. (Somerville, Massachusetts, USA) and two top parts that contain reservoirs and are made out of “HTM140” in the “Perfactory Micro HiRes” printer of Envisiontec GmbH (Gladbeck, Germany). The chip is clamped between both parts and the connection towards the top part is sealed with O-rings. The culture medium is in contact with the O-rings and the top holder structures. Therefore, non-cytotoxic O-rings out of E3609-70, which is a special ethylene propylene diene monomer rubber (EPDM), are purchased from Parker Hannifin GmbH (Kaarst, Germany). According to the manufacturer, this material passed the ISO 10993-5 and -10 as well as it is compliant to USP Class VI and USP <87> proving its biocompatibility and non-cytotoxicity.

**Figure 7:**
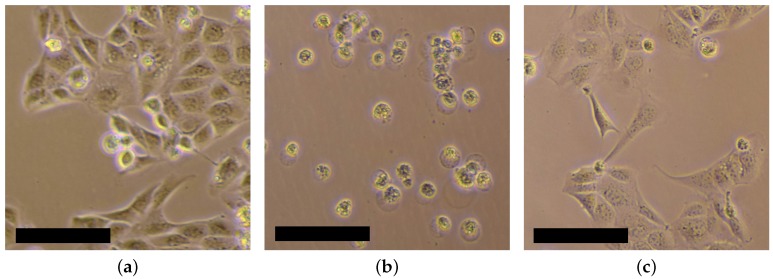
Culture of MDCK-cells with GMEM-medium 24 h after seeding: (**a**) negative sample without any 3D-printed parts showing high cell viability; (**b**) cell culture in which a 3D-printed part out of HTM140 is inserted showing influence of the toxicity of the material; (**c**) cell culture with a 3D-printed part out of HTM140 that is coated with 10 µm Parylene C showing the same cell viability as the negative sample. Scale bar is 100 µm.

The resin “HTM140” is cytotoxic even after printing, curing and rinsing. MDCK-cells were cultured in GMEM-medium in which one printed part was inserted. All cells died within 24 h while the negative sample without a printed part showed a high cell viability (see Figure 7a,b). Therefore, the holders are coated with 10 µm Parylene C by vapor deposition in the depositioning machine LC 300 LV 30 of Plasma Parylene Systems GmbH (Rosenheim, Germany). Parylene C is a non cytotoxic material that forms a pinhole free layer. Furthermore, Parylene C also coats the closed microfluidic channels with a penetration depth of up to 50 times of the channel diameter [35]. As a consequence, the toxic effects of the printed parts are suppressed completely. The MDCK-cells show a high viability after 24 h if a printed part of “HTM140” with a coating of 10 µm Parylene C is inserted into the culture medium (see Figure 7c).

### 3.4. Preparation of the Devices

Prior to the cell cultivation, the chip, the O-rings and the top holders are cleaned and sterilized intensively with isopropanol and ethanol. Subsequently, the setup is assembled in a sterile environment to avoid any contamination. The hydrogel is prepared by mixing agarose in deionized water with a concentration of 10 $\frac{g}{L}$. The solution is heated up to 100 ${}^{\circ }C$ in order to sterilize the hydrogel and to melt the agarose. Afterwards, the solution is cooled down to 50 ${}^{\circ }C$ and approximately 6 µL are inserted in each hydrogel channel. By cooling the chip to room temperature, the agarose gels and forms the membrane.

## 4. Experiments and Results

**Figure 8:**
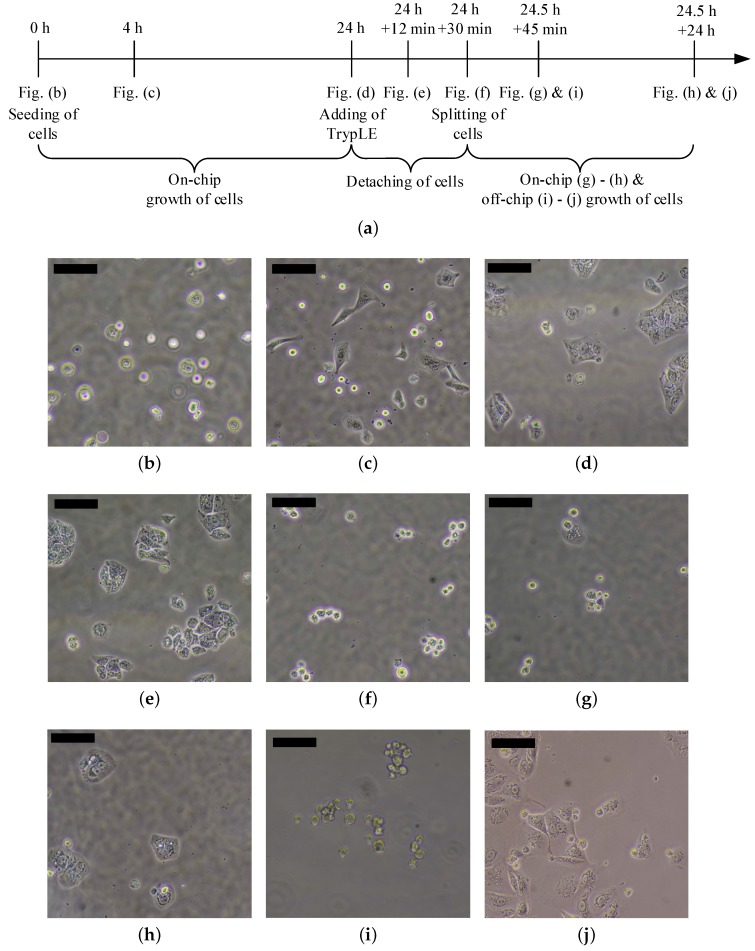
Culture of MDCK-cells (scale bar is 100 µm): (**a**) overview about the experimental procedure (not to scale); (**b**) t=0 h: seeding of the cells inside the chip; (**c**) t=4 h: cells adhere on the bottom plate; (**d**) t=24 h: cell growth inside the chamber; (**e**) t=24 h (culture) +12min incubation with TrypLE: first cells detach from the plate; (**f**) t=24 h+30min incubation with TrypLE: Detaching of almost all cells; (**g**) t=24.5 h+45min: A few cells remain inside the chip after the splitting; (**h**) t=24.5 h+24 h: on-chip growth of the cells; (**i**) t=24.5 h+45min: the majority of the cells is removed from the chip after splitting and added in a 24-well plate; (**j**) t=24.5 h+24 h: off-chip growth of the cells.

The procedure of the experiments is summarized in Figure 8a. After the preparation of the chip as described previously, MDCK-cells are inserted into the culture chamber (Figure 8b). After 4 h, the cells attach on the bottom plate and form their typical shape (Figure 8c). Within 24 h, a clear growth of the cells is observable (Figure 8d).

Subsequently, the medium in the culture chamber is replaced with TrypLE. TrypLE is a solution that contains cell-dissociation enzymes that detach mammalian cells. In contrast to Trypsin, TrypLE is more gentle and does not harm the cells in case of prolonged treatment. In addition, 12 min after adding TrypLE, the cells start to change their shape but do not detach yet (Figure 8e). Furthermore, 30 min after the treatment with the enzymes, the majority of the cells (here: approximately 90%) have a round shape, which shows that they are detached (Figure 8f) and these cells are removed by exchanging the liquid in the culture area. Subsequently, the removed cells are cultured off-chip in a 24-well plate. The remaining cells are cultured further inside the chip (Figure 8g 45 min after splitting). Here, a substantial cell growth is observable 24 h after splitting (i.e., 48.5 h after seeding, see Figure 8h). The cells that were transferred to the well-plate also show good viability and cell growth (45 min after splitting: Figure 8i and 24 h after splitting: Figure 8j).

## 5. Discussion

The presented device contains feeding structures for the supply with gases and nutrition. However, the entire assembly also has to be supplied from the macroscopic world. Due to the assembly structures with the reservoirs, the nutrition can be pipetted directly into the holder, which facilitate the use as no external equipment is required.

In contrast, the supply could be also automated by connecting pumps to the holders. The culture chamber is separated from the supply channels by agarose membranes. Therefore, the flow rate has no direct influence onto the growth (like it would have in perfusion based systems) as long as sufficient medium is pumped. Therefore, the requirements regarding the stability and the precision of the pump are very low, meaning that low cost pumps are suitable. Furthermore, even passive pumping that is low cost but usually provide non-constant flow might be used e.g., based on gravitational principles like a simple drip or capillary principles like absorption in paper-based microfluidics.

In the presented experiments, approximately 90% of the cells are removed with TrypLE. The number of cells that are detached depends on the duration of the treatment meaning that a longer treatment result in a higher ratio of detached cells. The number of the removed cells can be easily controlled by the time and observed by optical inspection.

All processes were executed on 100 mm wafer. Each chip has the outer dimensions of 13 mm by 17.5 mm so that each wafer contain 21 devices. The culture chamber, the hydrogel membranes and the supply channels cover only 25% of the chip surface showing the potential for further optimization of the design in order to reduce the chip size and thus the fabrication costs per chip.

The main requirements regarding the choice of materials are the non-cytotoxicity and the hydrophilic surfaces with the ability to pattern hydrophobic elements as phaseguides. Here, we chose silicon and glass, but these requirements are also fulfilled by low cost polymers like Cyclic olefin copolymer (COC) or Poly(methyl methacrylate) (PMMA). The hydrophobic surfaces are realized with ODT that adheres on gold as presented but also on copper [36], platinum or silver [37]. Platinum and gold are attractive for impedance measurements or cell manipulation like dielectrophoresis due to the good chemical stability. Applications might be impedance measurement of the cell culture or the control of the culture medium. Silver in combination with silver chlorid is often used for voltammetry or amperometry. Consequently, ODT allows the fabrication of the phaseguides out of the same material as the electrodes. However, other hydrophobic coatings such as hexamethyldisilazane (HMDS) or Parylene are also suitable. which shows that the presented concept is applicable to a variety of materials.

## 6. Conclusions

The presented microfluidic chip is an important step towards LoC-applications for the long-term on-chip cultivation of mammalian cells. For the first time, a microfluidic element contains two vertical membranes of agarose hydrogel to supply nutrition and gases to the cells. This supply of fresh medium and gases by diffusion result in a several advantages, namely:

- continuous but independent supply of gases and nutrients,
- low requirements of the pump regarding flow speed, precision and stability,
- no need for an external incubator,
- no shear stress onto the cells,
- the culture chamber could be closed once the cells are inserted resulting in a low contamination risk.

The comparison of the most important substances (glucose, oxygen, lactate and ammonium) shows that the oxygen has to be exchanged 10 times more frequently than the other compounds. Therefore, the supply of oxygen solely by diffusion limits the dimensions of the culture chamber. An analytical model is presented that allows to calculate the resulting oxygen profile and the maximal chamber width depending on the height, the membrane width and the filling factor. For the presented device, the growth chamber might be up to 3 mm wide if up to 50% of the area are covered with cells. The culture chamber is 8 mm long, so that the total area is 24 $mm^{2}$, which is 65% of the area of a single well in a 96-well plate and thus give space to a maximum of 30,000 cells.

The high diffusion rates benefit from the large cross-section area of the hydrogel due to the integration method based on the surficial phaseguides. Here, the filling of the melted hydrogel is controlled by flat elements on the bottom and top so that no obstacles like micropillars hamper the diffusion.

The chip is fabricated with standard clean room technologies and only non-cytotoxic materials like silicon or glass are used. The assembly that is used as connection to the macroscopic world is based on 3D printing. The experiments show that the printed parts are cytotoxic to mammalian cells, but this effect is eliminated by coating the surfaces with 10 µm Parylene C. Therefore, all materials that are in contact with the cells or the culture medium are non-cytotoxic and thus suitable for the long-term cultivation of mammalian cells.

Further experiments proved the on-chip cultivation of MDCK-cells. Here, the cells were seeded, fed with gases and nutrients over 24 h where cell growth was observable. Subsequently, the cells were detached with TrypLE, which took 30 min. A few cells remained in the chip and were cultivated for another 24 h. The majority of the detached cells was removed and cultured successfully in a multi-well-plate.

Because of the successful passaging including the seeding, feeding with oxygen and nutrients and removal of the cells, we conclude that the presented chip is suitable for the long-term cultivation of mammalian cells. The feeding structures for nutrients and gases are realized via vertical membranes next to the culture chamber. Furthermore, the device exhibit very good growth conditions as only non-cytotoxic materials are used for the chip but also for the connections to the macroscopic world. Furthermore, the concept enables the integration of whole cell-based sensors for the optical, electrical or chemical analysis. Last but not least, the requirements for the external supply elements (e.g., pumps) are very low and thus allow the use of Lab-on-a-Chip applications.

## Figures and Tables

**Table 1:** Comparison of different Lab-on-a-chips (LoC) for the long-term cultivation of mammalian cells.

Description	Year	Comment	Nutrient Supply	Gas Supply	Ref.
Blood-brain-barrier	2012	Coculture and conductivity measurements	Perfusion and diffusion through membrane	Diffusion through PDMS	[2]
PDMS-hydrogel hybrid reactor	2013	3D-culture on multi-electrode array	Diffusion through 3D-culture	Diffusion through PDMS	[3]
Lung cancer chip	2016	3D-2D-coculture	Perfusion, diffusion through 3D-culture	Diffusion through PDMS	[5]
3D-cell culture	2007	-	Diffusion through 3D-culture	Diffusion through PDMS	[6]
Breast-cancer analysis	2015	3D-cell culture	Diffusion through 3D-culture	Diffusion through PDMS	[7]
Blood-vessel- on-a-chip	2013	3D-Coculture	not possible	Diffusion through PDMS	[8]
Liver-on-a-chip	2016	Modular and pumpless platform for 2D-3D-coculture and electrical measurements	Perfusion and diffusion through 3D-culture	Oxygen dissolved in medium	[9]
Cell invasion	2016	Measurement of cell movement through membrane	Exchange of medium	Open reservoir	[12]
Integrated perfusion system	2011	Integration of heater, pump and electrical readout	Perfusion	Diffusion through PDMS	[13]
Shear-stress culture	2014	Chamber with different shear stress on cells	Perfusion	Diffusion through PDMS	[15]
Assay for coculturing	2012	Suitable for 2D and 3D-cell cultures	Diffusion through hydrogel	Diffusion through PDMS	[16]
Micro-lung	2017	Integration of membrane and electrical readout	Perfusion	Diffusion through PDMS	[17]
3D-cell culture	2005	Close chip out of glass and silicon	Perfusion and diffusion through 3D-culture	perfusion with fresh medium	[18]
Cancer metastasis under hypoxia	2014	Integration of gas supply and oxygen sensor	Perfusion	Diffusion through PDMS	[20]
CO_2_-control culture	2011	Gradient of CO_2_	Perfusion	Diffusion through PDMS	[21]
Hypoxia monitoring of cells	2015	Oxygen control and sensing for 3D-cell cultures	Perfusion	Diffusion through PDMS	[22]
On-chip incubator	2014	Integration of oxygen supply	Perfusion	Oxygen dissolved in medium	[23]
This work	2017	Platform for entire passaging process	Diffusion through hydrogel	Diffusion through hydrogel	-

PDMS: Polydimethylsiloxane.

**Table 2:** Comparison of the some compounds that are either consumed or produced by the cell. All values are given for MDCK-cells and are calculated for a cell density of $1\times 10^{6}\frac{\mathrm{cell}}{\mathrm{mL}}$.

Compound	Metabolic Rate	Concentration	Exchange Time	Diffusion Coefficient
Glucose	250 $\frac{\mathrm{amol}}{\mathrm{cell}\;s}$	25 mM	28 h	$2.4\times 10^{-9}\frac{m^{2}}{s}$
Lactate	490 $\frac{\mathrm{amol}}{\mathrm{cell}\;s}$	20 mM	11 h	$1\times 10^{-9}\frac{m^{2}}{s}$
Ammonium	11 $\frac{\mathrm{amol}}{\mathrm{cell}\;s}$	2 mM	50 h	$1.9\times 10^{-9}\frac{m^{2}}{s}$
Oxygen	20 $\frac{\mathrm{amol}}{\mathrm{cell}\;s}$	0.2 mM	2.7 h	$2.4\times 10^{-9}\frac{m^{2}}{s}$

## Abbreviations

The following abbreviations are used in this manuscript:

DRIE	Deep reactive ion etching
FEM	Finite-element-method
GMEM	Glasgow Minimum Essential Medium
LoC	Lab-on-a-chip
MDCK	Madin Darby Canine Kidney cell line
OCR	Oxygen consumption rate
ODT	Octadecanethiol
PDMS	Polydimethylsiloxane

## References

[1] Chin C.D., Linder V., Sia S.K. (2012). Commercialization of microfluidic point-of-care diagnostic devices. Lab Chip.

[2] Booth R., Kim H. (2012). Characterization of a microfluidic in vitro model of the blood-brain barrier. Lab Chip.

[3] Schurink B., Luttge R. (2013). Hydrogel/poly-dimethylsiloxane hybrid bioreactor facilitating 3D cell culturing. J. Vac. Sci. Technol. B Nanotechnol. Microelectron. Mater. Process. Meas. Phenom..

[4] Grist S.M., Chrostowski L., Cheung K.C. (2010). Optical Oxygen Sensors for Applications in Microfluidic Cell Culture. Sensors.

[5] Yu T., Guo Z., Fan H., Song J., Liu Y., Gao Z., Wang Q. (2016). Cancer-associated fibroblasts promote non-small cell lung cancer cell invasion by upregulation of glucose-regulated protein 78 (GRP78) expression in an integrated bionic microfluidic device. Oncotarget.

[6] Toh Y.C., Zhang C., Zhang J., Khong Y.M., Chang S., Samper V.D., van Noort D., Hutmacher D.W., Yu H. (2007). A novel 3D mammalian cell perfusion-culture system in microfluidic channels. Lab Chip.

[7] Jeon J.S., Bersini S., Gilardi M., Dubini G., Charest J.L., Moretti M., Kamm R.D. (2014). Human 3D vascularized organotypic microfluidic assays to study breast cancer cell extravasation. Proc. Natl. Acad. Sci. USA.

[8] Van der Meer A.D., Orlova V.V., ten Dijke P., van den Berg A., Mummery C.L. (2013). Three-dimensional co-cultures of human endothelial cells and embryonic stem cell-derived pericytes inside a microfluidic device. Lab Chip.

[9] Esch M.B., Ueno H., Applegate D.R., Shuler M.L. (2016). Modular, pumpless body-on-a-chip platform for the co-culture of GI tract epithelium and 3D primary liver tissue. Lab Chip.

[10] Jin H., Yu Y. (2016). A Review of the Application of Body-on-a-Chip for Drug Test and Its Latest Trend of Incorporating Barrier Tissue. J. Lab. Autom..

[11] Skardal A., Shupe T., Atala A. (2016). Organoid-on-a-chip and body-on-a-chip systems for drug screening and disease modeling. Drug Discov. Today.

[12] Lei K.F., Tseng H.P., Lee C.Y., Tsang N.M. (2016). Quantitative Study of Cell Invasion Process under Extracellular Stimulation of Cytokine in a Microfluidic Device. Sci. Rep..

[13] Lin J.L., Wang S.S., Wu M.H., Oh-Yang C.C. (2011). Development of an Integrated Microfluidic Perfusion Cell Culture System for Real-Time Microscopic Observation of Biological Cells. Sensors.

[14] Stolberg S., McCloskey K.E. (2009). Can shear stress direct stem cell fate?. Biotechnology Progress.

[15] Hattori K., Munehira Y., Kobayashi H., Satoh T., Sugiura S., Kanamori T. (2014). Microfluidic perfusion culture chip providing different strengths of shear stress for analysis of vascular endothelial function. J. Biosci. Bioeng..

[16] Shin Y., Han S., Jeon J.S., Yamamoto K., Zervantonakis I.K., Sudo R., Kamm R.D., Chung S. (2012). Microfluidic assay for simultaneous culture of multiple cell types on surfaces or within hydrogels. Nat. Protoc..

[17] Noh S., Kim H. A micro lung chip to assess air pollutant effects. Proceedings of the IEEE 30th International Conference on Micro Electro Mechanical Systems (MEMS).

[18] Frisk T., Rydholm S., Andersson H., Stemme G., Brismar H. (2005). A concept for miniaturized 3-D cell culture using an extracellular matrix gel. Electrophoresis.

[19] Brennan M.D., Rexius-Hall M.L., Elgass L.J., Eddington D.T. (2014). Oxygen control with microfluidics. Lab Chip.

[20] Acosta M.A., Jiang X., Huang P.K., Cutler K.B., Grant C.S., Walker G.M., Gamcsik M.P. (2014). A microfluidic device to study cancer metastasis under chronic and intermittent hypoxia. Biomicrofluidics.

[21] Forry S.P., Locascio L.E. (2011). On-chip CO_2_ control for microfluidic cell culture. Lab Chip.

[22] Grist S., Schmok J., Liu M.C., Chrostowski L., Cheung K. (2015). Designing a Microfluidic Device with Integrated Ratiometric Oxygen Sensors for the Long-Term Control and Monitoring of Chronic and Cyclic Hypoxia. Sensors.

[23] Takano A., Tanaka M., Futai N. (2014). On-chip multi-gas incubation for microfluidic cell cultures under hypoxia. Biomicrofluidics.

[24] Berthier E., Young E.W.K., Beebe D. (2012). Engineers are from PDMS-land, Biologists are from Polystyrenia. Lab Chip.

[25] Regehr K.J., Domenech M., Koepsel J.T., Carver K.C., Ellison-Zelski S.J., Murphy W.L., Schuler L.A., Alarid E.T., Beebe D.J. (2009). Biological implications of polydimethylsiloxane-based microfluidic cell culture. Lab Chip.

[26] Sackmann E.K., Fulton A.L., Beebe D.J. (2014). The present and future role of microfluidics in biomedical research. Nature.

[27] Volpatti L.R., Yetisen A.K. (2014). Commercialization of microfluidic devices. Trends Biotechnol..

[28] Bunge F., van den Driesche S., Vellekoop M.J. A novel on-chip element to provide mammalian cell cultivation and passaging to labs-on-chip. Proceedings of 19th International Conference on Solid-State Sensors, Actuators and Microsystems (TRANSDUCERS).

[29] Bunge F., van den Driesche S., Vellekoop M.J. (2016). Symmetric surficial phaseguides: A passive technology to generate wall-less channels by two-dimensional guiding elements. Microfluid. Nanofluid..

[30] Vulto P., Podszun S., Meyer P., Hermann C., Manz A., Urban G.A. (2011). Phaseguides: A paradigm shift in microfluidic priming and emptying. Lab Chip.

[31] Mot A.I., Liddell J.R., White A.R., Crouch P.J. (2016). Circumventing the Crabtree Effect: A method to induce lactate consumption and increase oxidative phosphorylation in cell culture. Int. J. Biochem. Cell Biol..

[32] Sidorenko Y., Wahl A., Dauner M., Genzel Y., Reichl U. (2008). Comparison of Metabolic Flux Distributions for MDCK Cell Growth in Glutamine- and Pyruvate-Containing Media. Biotechnol. Prog..

[33] Altamirano C., Berrios J., Vergara M., Becerra S. (2013). Advances in improving mammalian cells metabolism for recombinant protein production. Electron. J. Biotechnol..

[34] Wagner B.A., Venkataraman S., Buettner G.R. (2011). The rate of oxygen utilization by cells. Free Radic. Biol. Med..

[35] Flueckiger J., Bazargan V., Stoeber B., Cheung K.C. (2011). Characterization of postfabricated parylene C coatings inside PDMS microdevices. Sens. Actuators B Chem..

[36] Ron H., Cohen H., Matlis S., Rappaport M., Rubinstein I. (1998). Self-Assembled Monolayers on Oxidized Metals. 4. Superiorn-Alkanethiol Monolayers on Copper. J. Phys. Chem. B.

[37] Li Z., Chang S.C., Williams R.S. (2003). Self-Assembly of Alkanethiol Molecules onto Platinum and Platinum Oxide Surfaces. Langmuir.

